# From Discovery to Delivery: A Rapid and Targeted Proteomics Workflow for Monitoring Chinese Hamster Ovary Biomanufacturing

**DOI:** 10.1016/j.mcpro.2025.101011

**Published:** 2025-06-04

**Authors:** Charles Eldrid, Ellie Hawke, Kathleen M. Cain, Kate Meeson, Joanne Watson, Reynard Spiess, Luke Johnston, William Smith, Matthew Russell, Robyn Hoare, John Raven, Jean-Marc Schwartz, Magnus Rattray, Leon Pybus, Alan Dickson, Andrew Pitt, Perdita Barran

**Affiliations:** 1 Manchester Institute of Biotechnology, University of Manchester, Manchester, UK; 2 Institute of Quantitative Biology, Biochemistry & Biotechnology, School of Biological Sciences, University of Edinburgh, Edinburgh, UK; 3 FUJIFILM Diosynth Biotechnologies, Billingham, UK; 4 Faculty of Biology, Medicine and Health, University of Manchester, Manchester, UK

**Keywords:** biotechnology, CHO cell biology, proteomics, quantitative proteomics, multiple reaction monitoring

## Abstract

Chinese hamster ovary (CHO) cells are the industrial workhorse for manufacturing biopharmaceuticals, including monoclonal antibodies. CHO cell line development requires a more data-driven approach for the accelerated identification of hyperproductive cell lines. Traditional methods, which rely on time-consuming hierarchical screening, often fail to elucidate the underlying cellular mechanisms driving optimal bioreactor performance. Big data analytics, coupled with advancements in “omics” technologies, are revolutionizing the study of industrial cell lines. Translating this knowledge into practical methods widely utilized in industrial biomanufacturing remains a significant challenge. This study leverages discovery proteomics to characterize dynamic changes within the CHO cell proteome during a 14-day fed-batch bioreactor cultivation. Utilizing a global untargeted proteomics workflow on both a ZenoTOF 7600 and a Cyclic IMS QToF, we identify 3358 proteins and present a comprehensive data set that describes the molecular changes that occur within a well-characterized host chassis. By mapping relative abundances to key cellular processes, eight protein targets were selected as potential biomarkers. The abundance of these proteins through the production run is quantified using a 15-min targeted triple quadrupole (MRM) assay, which provides a molecular-level QC for cell viability. This discovery to target workflow has the potential to assist engineering of new chassis and provide simple readouts of successful bioreactor batches.

## Highlights

• Understanding the host chassis for biologic production is key for the development of biotechnology.
• Discovery proteomics was used to identify cellular changes within Chinese Hamster Ovary cells under bioreactor conditions.
• Discovery results were translated into a targeted mass spectrometry which can be used to monitor host cells under bioreactor conditions.
• The biological understanding gained from the discovery of proteomics highlights possible methods of increasing bioreactor performance while identifying possible gene engineering targets.

## Graphical abstract

**Figure:**
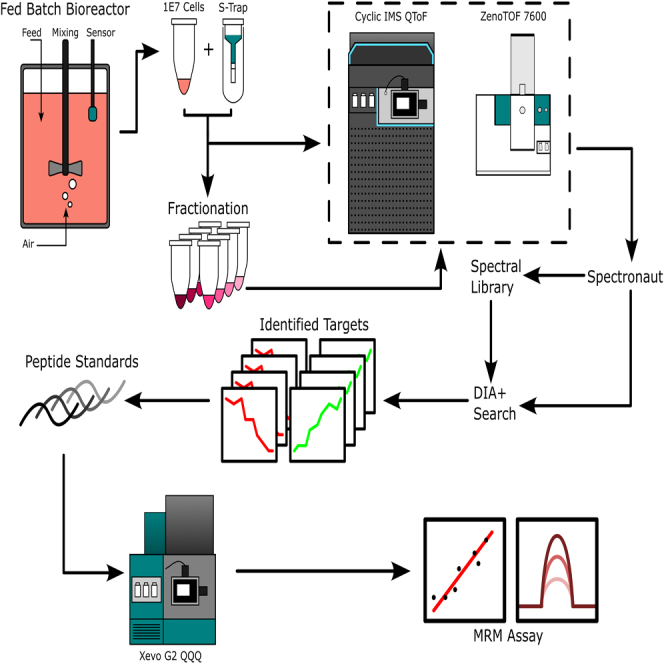

## In Brief

Chinese Hamster Ovary cells are the main chassis for the production of biologics such as monoclonal antibodies. Understanding their cellular processes under bioreactor conditions is important for the development of more efficient cell lines. We use discovery proteomics to identify key molecular pathways being regulated throughout a bioreactor run. This was translated into a targeted assay for monitoring bioreactors, and identifies possible methods for the improvement of biologics production.

Chinese hamster ovary (CHO) cells are the predominant host cell for recombinant biopharmaceutical manufacturing (1, 2). Biotherapeutics or biologics encompass any therapeutic derived from biological sources. The global biologics market in 2024 was valued at $458 billion, with monoclonal antibodies (mAbs) comprising the largest segment at ∼$263 billion (3, 4), (https://www.statista.com/statistics/280578/global-biologics-spending/), (https://www.fortunebusinessinsights.com/monoclonal-antibody-therapy-market-102734).

CHO cell lines have become the workhorse for biotherapeutic manufacturing due to several key advantages. Their ability to grow in suspension culture using serum-free, chemically-defined media, combined with robust selection/amplification expression systems and their capacity to perform “human-like” post-translational modifications (PTMs), has solidified their role in the manufacture of life-changing medicines. Industrially optimized expression platforms routinely achieve mAb titers of up to 10 g/L in fed batch cultures with dramatically reduced development timelines (1, 5, 6, 7, 8, 9)

Improvements in productivity and quality of host cell lines have been made through “brute force” modification and screening of media and feeds (10), and selection of host cell lines (11). However, efforts remain to transition CHO expression systems from reliable workhorses to state-of-the-art protein-producing powerhouses. CHO-derived mAbs have historically offered a low-risk development pathway that relies on platform expression, development, manufacturing technology, and infrastructure (9). The evolving biopharmaceutical landscape demands more flexibility (12). Diversification of product types, from traditional mAbs to novel modalities, presents various challenges (13, 14, 15). Moreover, the imperative to reduce time, cost, and environmental impact necessitates innovative new approaches.

Despite significant advancements in CHO expression systems, a fundamental understanding of the underlying cellular mechanisms driving improved bioprocess performance remains elusive. This knowledge gap often necessitates time-consuming and resource-intensive empirical optimization. The development of predictive biomarkers could significantly enhance biopharmaceutical development by enabling early-stage selection of high-performing cell lines and processes, thereby reducing the reliance on extensive empirical screening (16).

“Omics” technologies such as metabolomics and proteomics are widely employed in healthcare research, providing mechanistic insights into disease states and in turn identifying biomarkers and potential drug targets (17). If applied to a host cell system used to produce a given biologic, these methods would inform on the viability of the cells and the efficiency of how they produce the target product at a molecular level, throughout a batch production run typically of 2 weeks. Mass spectrometry-based proteomics could be used to optimize the design of host cell chassis, in the development of feedstocks, to increase product titer and improve cell viability. Despite this potential, adoption of discovery proteomics in industrial biotechnology is not common, perhaps hindered by the need for specialized instrumentation, highly skilled users, complex sample preparation and lengthy analysis. We believe that large-scale discovery proteomics on a given host cell would provide a rich reference dataset and a starting point for chassis optimization. Furthermore, results from discovery analysis could be translated into targeted assays, with simplified sample preparation, a yes/no readout and the capability for robust reproducible high-throughput analysis. This simple readout could provide reference data for regulatory compliance, analogous to the use of targeted mass spectrometry methods in environmental monitoring.

**Fig. 1:**
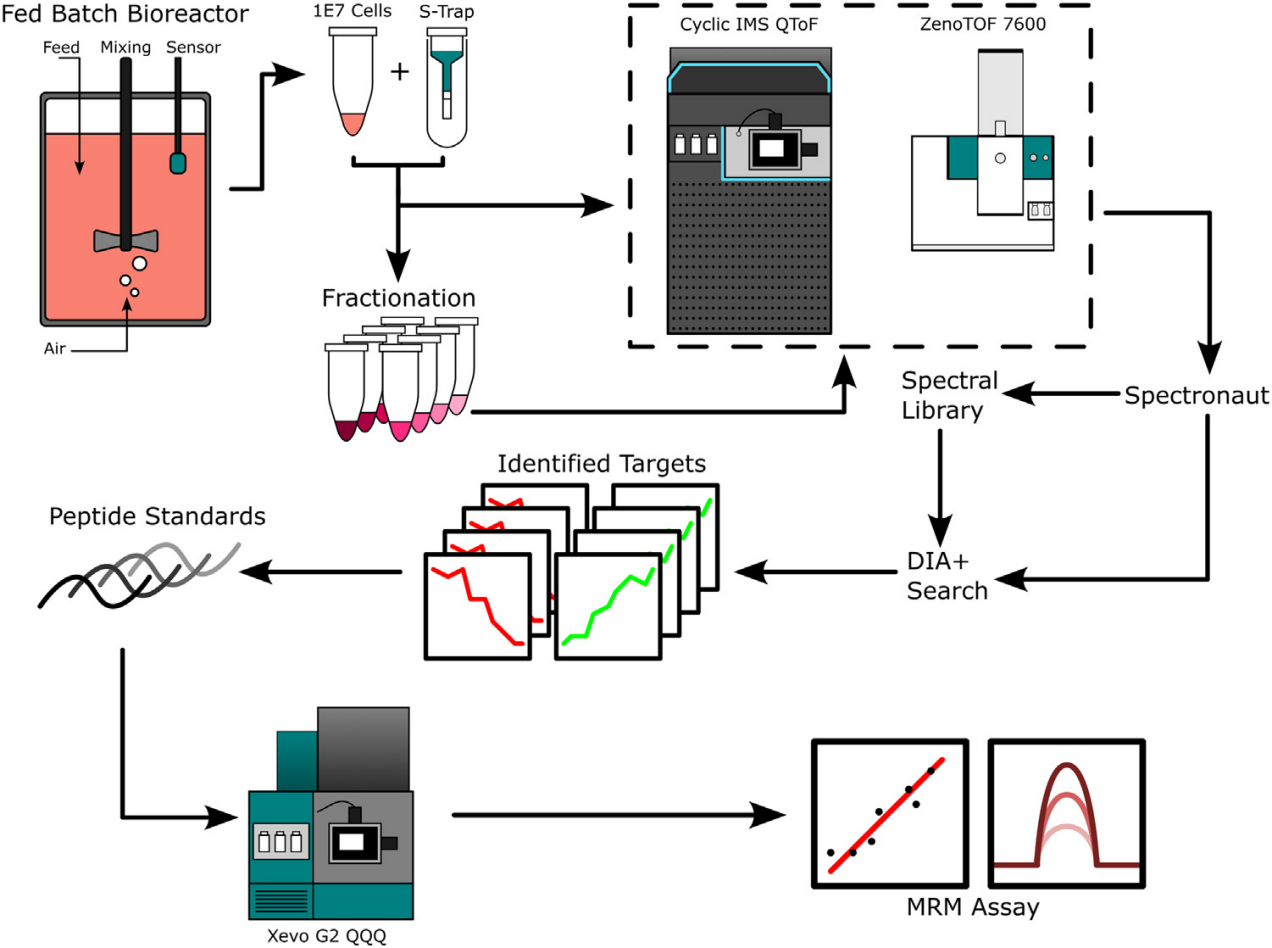
**Sample workflow, from bioreactor sampling to MRM assay.** Samples are taken from bioreactors and prepared using S-traps. Peptides from control cell pellets are fractionated to produce a spectral library. Global untargeted discovery data are collected on both a ZenoTOF 7600 and a Cyclic IMS QToF. Data are searched using Spectronaut, with a combined spectral library, DIA+ search to increase depth and confidence of identifications. Diagnostic peptides from protein targets are selected for translation into a targeted quantification study on an Xevo TQS. Relative quantities of proteins inform on the status of cells within the bioreactor.

To demonstrate this workflow (Fig. 1), we here apply discovery proteomics to analyze a CHO cell fed-batch bioreactor daily from days 0 to 14. Our results, based on quantifying more than 2500 proteins, reveal dynamic changes in the proteome throughout the bioreactor culture, allowing us to identify three distinct culture phases which map to “growth”, “stationary phase”, and “cell death” based on differential protein quantification. Following pathway analysis, we developed a 15-min targeted multiple reaction monitoring (MRM) assay for eight biomarkers, enabling more efficient analysis of the key phases of bioreactor cultivation in industrial settings.

## Experimental Procedures

For full methods and materials, including sample preparation, instrument parameters, and analysis parameters, please see the supplementary information.

### Fed Batch Bioreactors

The proprietary FUJIFILM Diosynth Biotechnologies (FDB) CHO DG44-IgG-expressing cell line was cultured in FDB-MAP (Sigma) medium supplemented with 175 nM MTX (Sigma) and 8 mM L-glutamine (Sigma). For maintenance culture, the cell line was subcultured every 3 to 4 days in vented Erlenmeyer shake flasks (Corning) at a seeding density of 0.5 x 10^6^ cells/ml and maintained in a shaking cell culture incubator. Cultures were expanded to attain the required numbers prior to seeding bioreactors.

Triplicate fed-batch bioreactor cultures were performed over a 14-day period in 2 L single-use Univessel'(R) (Sartorius). Culture medium for 2 L bioreactor fed-batch cultures was JM-05B (FUJIFILM Irvine Scientific), supplemented with 8 mM L-glutamine. The bioreactors were sampled immediately after inoculation and daily for viable cell count (VCC), viability, extracellular metabolite concentrations, and samples for proteomic analysis. Bioreactor sampling preceded daily bioreactor feeding, with cultures fed daily from day 2 onward using a proprietary feeding regime.

### Proteomics Sample Preparation

Cell pellets (1 x 10^7^ cells) were prepared according to the S-trap protocol (18). Briefly, cells were lysed in a 10% (w/v) SDS lysis solution, and sonicated. To remove DNA, 2 μl Benzonase (Cambridge Bioscience Limited) were added, and the samples were sonicated again. Samples were clarified by centrifugation and protein concentration of lysates was determined using the Pierce BCA Protein Assay Kit (ThermoFisher Scientific). For each sample 30 μg protein was reduced using TCEP (Cambridge Bioscience Limited) for 15 min at 55 °C and alkylated using 20 mM IAA (Fluorochem Limited) for 1 h in the dark at room temperature, then 2.5 μl phosphoric acid (VWR International Ltd) was added. Protein was precipitated through the addition of 165 μl binding/wash buffer and applied to S-Trap (18) micro spin columns (VWR International). Sample were washed three times with 150 μl binding/wash buffer, and 1:10 ratio of trypsin gold (Promega) to protein was added to the S-trap and incubated overnight at 37 °C in a thermomixer (Starlabs). After incubation, peptides were eluted with 50 μl of the appropriate elution buffers. The eluted solution was dried using an Eppendorf concentrator plus before being resuspended in 0.1% (v/v) formic acid before MS analysis. Fractionated peptides for spectral library creation were prepared by applying 100 μg peptide mixtures harvested from samples of cell pellets collected from maintenance sub-culture flasks (equivalent to exponential phase in bioreactor culture) to Pierce high pH reversed-phase peptide fractionation spin columns and prepared according to protocol (ThermoFisher Scientific).

### LC-MS

200 ng of peptides were directly injected onto a nanoEase M/Z Peptide CSH C18 Column, 130 Å, 1.7 μm, 300 μm × 150 mm (Waters Corp) on a Acquity UPLC M-class system. ZenoTOF 7600 data were collected in positive mode using a 50-min gradient at 2 μl/min (see Supplemental Table S1), with an OptiFlow 50 to 200 μl Micro/MicroCal source. Autocalibration was performed every five injections. For data-independent acquisition (DIA) mode instrument parameters (Supplemental Table S2), data were collected using 105 variable windows from 450 to 2050 m/z (Supplemental Table S3), for spectral library creation, data-dependent acquisition (DDA) was used (Supplemental Table S4). For cyclic IMS QToF, data were collected in DIA mode with a 100-min gradient (Supplemental Table S6), with mobility, using system settings adapted from Nagy, K. *et al*. (19) (Supplemental Tables S7 and S8).

### MRM Assay Development

To develop a tier three multiple reaction monitoring assay, synthetic target peptides were purchased from JPT Peptide Technologies (DE), prepared in 0.1% (v/v) formic acid, and used for method development. Data were collected using a 15 min gradient (Supplemental Table S9) on an Xevo TQS (Waters) triple quad mass spectrometer. Targeted method development, initial instrument acquisition file creation, collision energy optimization and fragment selection were all performed using Skyline (v23.1.0.455) (Supplemental Tables 10 and S11). “Quantotypic” target peptides were selected, which were found to be unique to the mammalian Uniprot database.

### Data Analysis

Data were analyzed using Spectronaut v18.4 (Biognosys) (20) using BGS factory settings in a peptide-centric fashion. A spectral library was created from fractionated peptide DDA using Pulsar, generated from an *in-silico* library based on mammalian protein sequences from Uniprot. DIA data were searched using the spectral library and the mammalian sequences. Uniprot accessions were mapped to gene names using the Uniprot ID mapper tool (https://www.uniprot.org/id-mapping). Missing gene names not assigned by the tool were mapped manually. Statistically enriched pathways were identified using Reactome (21).

### Experimental Design and Statistical Rationale

Data for each biological replicate was collected once on each instrument. Proteins that had a Q-value of below 0.05 and displayed an absolute average log fold change value of above 0.75 between the two conditions were highlighted as targets.

## Results and Discussion

### Fed Batch Bioreactor

**Fig. 2:**
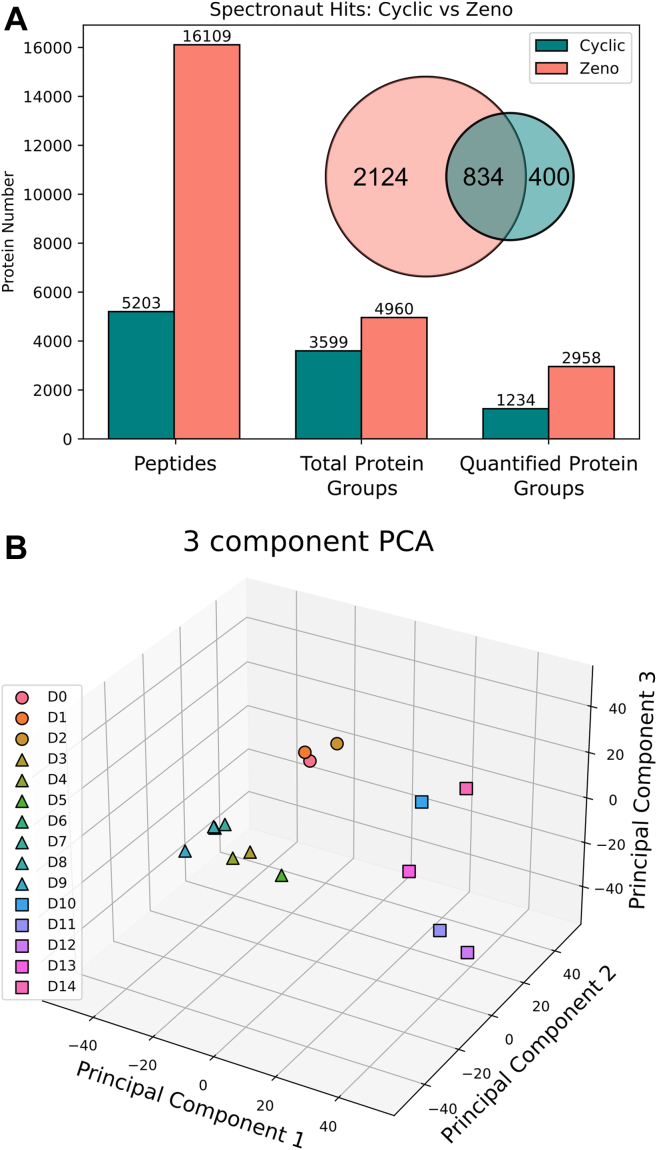
**Discovery proteomics results.***A*, total protein group hits collected from both ZenoTOF 7600 (*pink*) and Cyclic IMS QToF (*green*), with the overlap in identified and quantified protein groups between both groups as a Venn diagram (inset). *B*, unsupervised PCA analysis of the global proteomics quantification from day 0 (seeding) to day 14 (harvesting), with the three culture phases: growth (*circles*), stationary phase (*triangles*), and cell death (*squares*).

One sample per day was taken from triplicate fed batch bioreactors, over a 14-day production from inoculation (day 0) to harvest (day 14), run under standard platform process conditions. Each sample was analyzed independently by two mass spectrometers: a cyclic IMS QToF (Waters Corp, UK) and a ZenoTOF 7600 (SCIEX, UK). 3599 proteins were identified on the Cyclic IMS QToF, 1234 of which were quantified across 14 days. On the ZenoTOF 7600, 4960 proteins were identified, with 2958 quantified (see Fig. 2*A*), an increase of approximately x2.4. Of the quantified proteins identified as having differential abundance, 834 proteins were found to be in common, which is ∼2/3 of the total identified with the Cyclic. Identified proteins may include some variants, but are grouped to remove redundancies.

**Fig. 3:**
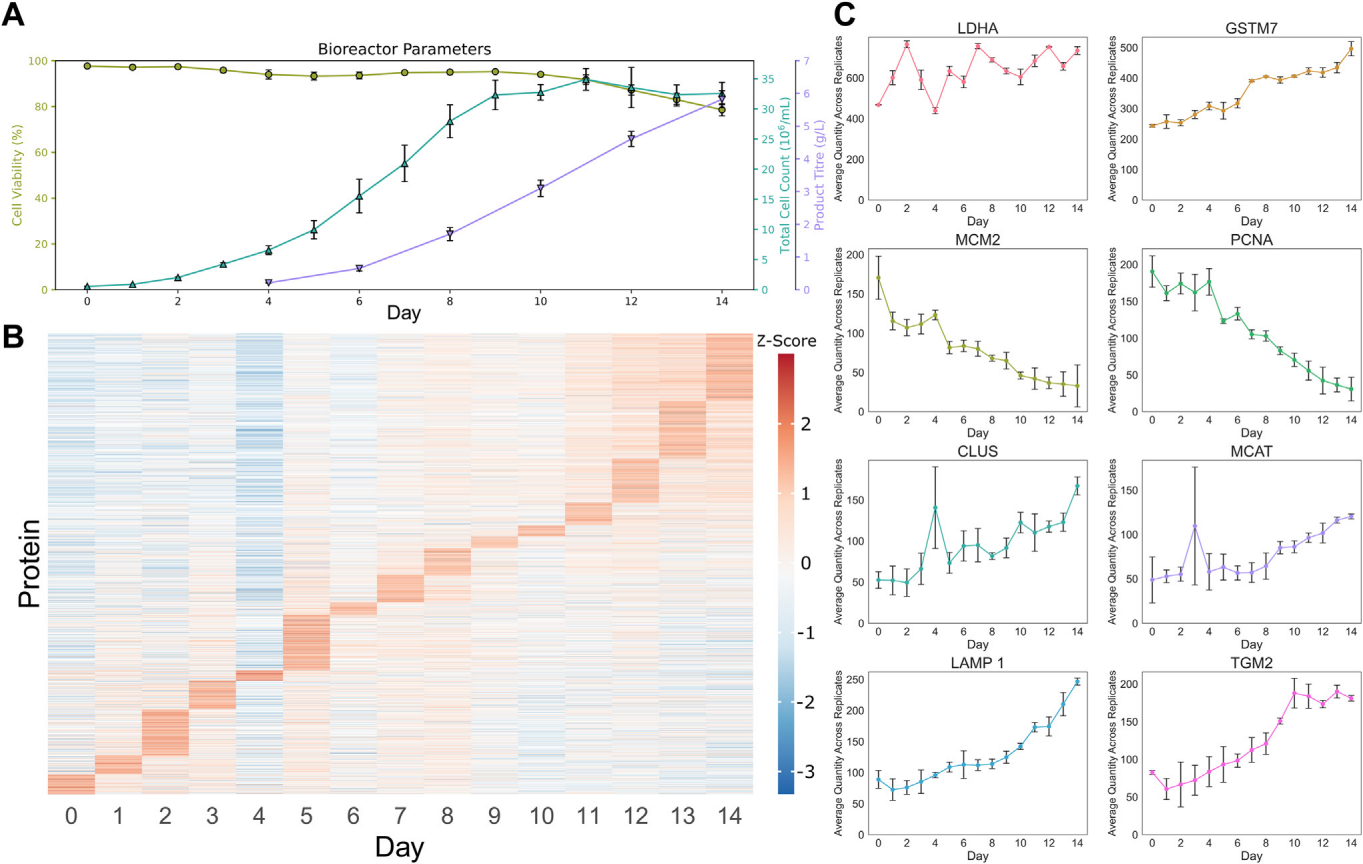
**Observed changes under production*****conditions.**A*, gross bioreactor parameters recorded by Biostat: cell viability, total cell count, and product titre based on three separate bioreactor runs. *B*, quantified proteins arranged by Z-score, showing continual development of the proteome across the bioreactor run from day 0 (*seeding*) to day 14 (*harvesting*). *C*, quantification profiles of protein targets that were observed to undergo significant change in LFC and were selected for targets in the MRM assay.

Principal component analysis (PCA) clusters the bioreactor runs into three separate groups using three components, which describes 55% of the variance: days 0 to 3, days 4 to 9, and days 10 to 14 (see Fig. 2*B*). These groupings correlate with culture phases identified from bioreactor stats such as viable cell count and total cell count (Fig. 3*A*) and are well described in the literature (22). Broadly, these bioreactor phases correlate with different cell states, namely: cellular growth, stationary phase, and cell death. We observe a sigmoidal curve of total cell count, with proliferation occurring steadily between days 0 to 8 and cell viability remaining high until day 10, where it begins to drop as the cells enter the stress phase. Product titer is measurable from day 4 and increases steadily until harvesting on day 14.

While the PCA shows grouping into three broad metabolic states, there is a clear observable progression of the protein expression, where each day has a set of proteins which are most abundant (Fig. 3*B*). There is a distinct drop in the Z-score of multiple proteins on day 4, highlighting this point as a key shift in the bioreactor between culture phases, previous work within this system has identified this day where the “lactate switch” occurs, moving from production to consumption of lactate (23). Pathway analysis of the data revealed that pathways relating to proliferation, DNA replication, and energy production changed significantly between key time points (days 1, 4, 7, and 14), which cover the metabolic transitions between the culture phases (see SI). Proteins that relate to these pathways were observed to undergo high log fold change (LFC) across the bioreactor runs, and were identified as being of high importance through prior knowledge of CHO cell metabolism, and were selected as targets (Fig. 3*C*) (22, 24).

Eight proteins (Fig. 3*C*), of which seven were observed with both instruments, were selected for targeted assays which will then be representative of the global changes that occur in the bioreactor across batch production. The chosen peptides are from the following key proteins that have been shown to differentially regulate across production: DNA replication licensing factor MCM2 (MCM2; Uniprot ID: P49736), proliferating cellular nuclear antigen (PCNA; Uniprot ID: P57761) inform on cell proliferation (25, 26, 27); lactate dehydrogenase chain A (LDHA; Uniprot ID: P06151) and mitochondrial carnitine/acylcarnitine transferase (MCAT; Uniprot ID: Q9Z2Z6), provide insights to energy production (23, 28, 29, 30); Glutathione S-transferase Mu 7 (GSTM7; Uniprot ID: P08009) and lysosome associated membrane protein 1 (LAMP1; Uniprot ID: P49129), both report on the effects of oxidative stress and autophagy (31, 32, 33); Protein-glutamine gamma-glutamyltransferase 2 (TGM2; Uniprot ID: P21981) plays a key role in apoptosis as well as other processes (34, 35, 36). Additionally Clusterin (CLUS; Uniprot ID: P05371), a chaperone, was chosen since it is commonly observed co-eluting with mAbs during protein-A purification and binds non-specifically to IgG Fab and Fc regions (37, 38).

The rationale for choosing these proteins is that they all represent different critical processes during the culture phase and follow observable trends of quantification. Proteins corresponding to growth and proliferation (MCM2, PCNA) reduce across the bioreactor run as cells shift from growth to production. LDHA appears to undergo a sharp increase during the growth phase, before dropping to its lowest point at day 4, where the lactate switch occurs, before increasing back to day 2 levels at day 8 with some fluctuations throughout the rest of the run. CLUS follows a steady increase throughout the run, but with a spike at day 4, highlighting this as a key change point in the bioreactor phase. CLUS is an extracellular chaperone that has a broad effect on preventing the misfolding of proteins (37, 39); however, when intracellular, it is thought to have an anti-apoptotic function, protecting cells from misfolded-protein response (40). It is a commonly observed co-purified host cell protein (HCP) (38, 41) and considering the specific observations of CLUS binding IgG (37) it is a marker for the production of folded mAb in CHO. MCAT increases steadily, with high levels of variability between bioreactors on day 3, prior to the lactate switch. MCAT shuttles carnitine between layers of the mitochondrial membrane and is essential for fatty acid oxidation within the mitochondria and production of cellular energy (28, 29). However, carnitine transport also supports the lactate switch, amino acid production, and buffers oxidative stress in CHO cells, highlighting its importance in monitoring its action (42, 43). TGM2 increases steadily throughout the run and plateaus between days 10 and 14 during the stress phase. TGM2 has a wide range of biological functions revolving around transamidase and isomerase activity (34, 35, 36, 44) and has been observed co-purified in CHO-produced mAb (45). While it has complex regulation and activity, it is involved in cellular signaling in response to inflammation and protein misfolding response; we can infer that this is a marker of cellular stress (36).

MRM assays are more robust and simpler and would provide a go-to method for routine use in industrial settings. Triple quadrupoles have higher sensitivity than discovery platforms, although here we have selected peptides where the abundance and change in abundance are well within the sensitivity of most modern LC-MS instruments.

**Fig. 4:**
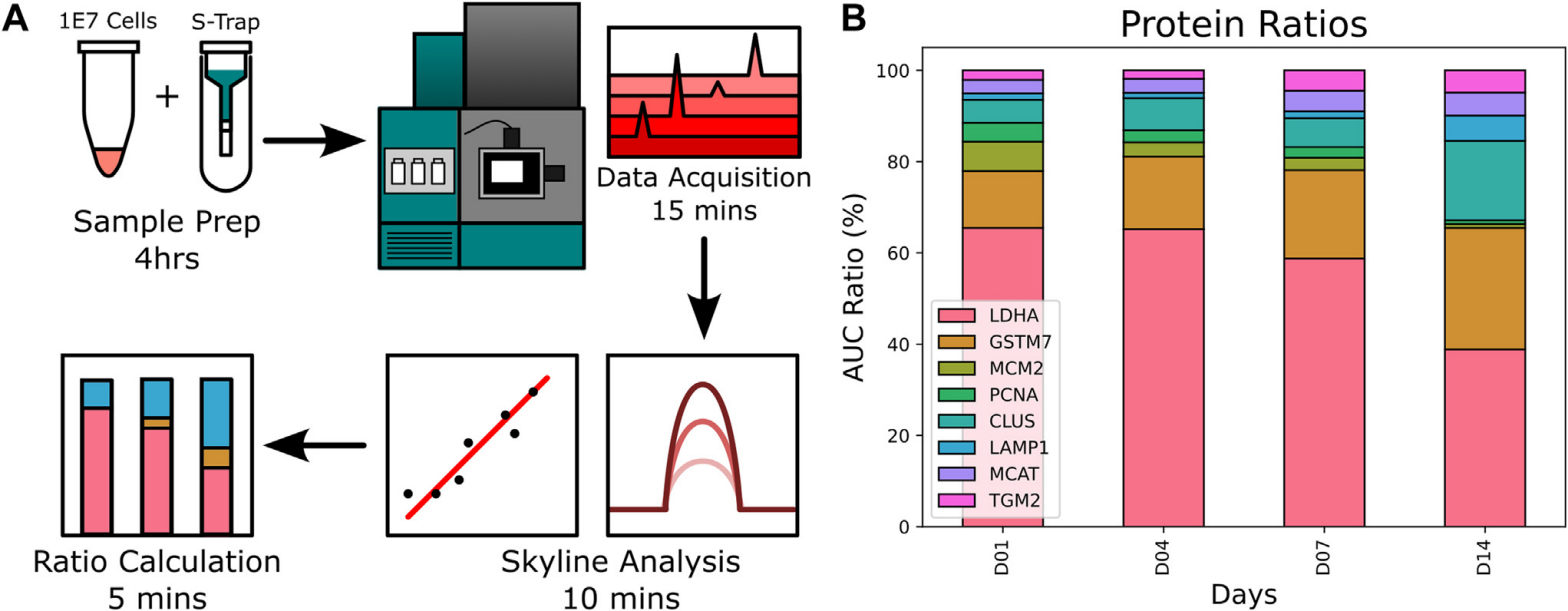
**MRM analysis**. *A*, workflow figure for MRM data collection: sample prep via S-trap, data collection, skyline analysis, and calculation of the relative ratios of identified biomarkers. *B*, ratio of area under the curve (AUC) of the selected protein targets quantified by MRM assay on key days within the bioreactor run.

Triplicate bioreactor samples were acquired using the developed MRM method. Higher variation in protein abundances was observed within the MRM assay examining each bioreactor separately (Supplemental Fig. S1) than with global untargeted methods (Fig. 3*C*). In our discovery proteomics workflow such variation is reduced during data normalization steps. Despite this, observed trends in protein abundance were consistent between bioreactors (Supplemental Fig. S2), and the use of peptide standards mean that ratios of protein abundance within the bioreactor can be applied to identify the cellular phase. Comparison of ratios of peptide from the target proteins from four different days during the bioreactor process allows us to normalize for the variation between bioreactors. The ratios of the quantified proteins give an understanding of the bioreactor state. LDHA, while dominating over 60% of the quantification, drops by 20% by day 14, allowing proteins such as GST7 and CLUS to become more dominant, forming approximately 40%. MCM2 and PCNA become negligible by day 14, whereas before they comprised 10% of the signal. Proteins such as TGM2 and MCAT increase to become about 10% of the total signal. This simple assay now provides clear evidence of the change in abundance (Fig. 4). From MRM measurements of each bioreactor these ratios of peptides can quantify expression changes in these key proteins. This approach, using data from 12 separate injections (total machine time ∼3 h), can be readily analyzed in minutes to provide robust quantification of pathways that are altered in the bioreactors.

## Conclusion

Discovery proteomics is a powerful tool for understanding cellular biology, supplying information on cellular status through the identification and quantification of proteins (46). The physical, analytical, and computational work required for the discovery of quantitative proteomics means that it is not suitable for routine analysis. Translating discovery proteomics results into a targeted MRM assay provides exquisite detail on altered pathways in a simpler assay. By taking this approach, we can report on key processes that in turn inform on the status of bioreactors during production of mAb from CHO cells. In doing this, we translate biological understanding gained from discovery proteomics into a rapid, quantitative, and simple-to-perform assay with easily read data. Comparison of simple ratios of protein quantities gives a clear indication of cellular metabolism, cell growth, stress, and cell death. This assay, which could be plate-based, can require as little as 4 h of sample preparation (18) and 15 min of machine time per sample, providing accessible data within minutes.

This method has advantages over measuring viable cell count and other bioreactor parameters as it reports on the underlying biology of the culture phase through the quantitation of protein biomarkers, i.e., oxidative stress can be diagnosed as a cause of drop in cell viability, rather than simply reporting a lowering VCC. While immunoassays such as Western blots and enzyme-linked immunosorbent assays (ELISAs) are classical techniques for protein quantification, the development of appropriate antibodies can be time-consuming, may display cross-reactivity, and have detection ranges within the nanogram levels. In comparison, MRM techniques are highly sensitive, specific and high throughput. They can be quickly adjusted to identify and quantify PTMs without the need for the creation of new antibodies. Costs of primers for assays such as reverse transcription-polymerase chain reaction (RT-PCR) are higher than for peptides, and are required for continued analysis, whereas synthetic peptides are only required for method development.

While proteomics requires offline digestion of proteins from cellular lysate in order to create the detectable peptides for MRM assays, quantification of specific proteins has advantages over monitoring via other omics techniques. Protein is ultimately the describer of phenotype: while transcriptomics gives a measurement of global genes expression there is not always a one-to-one relation between transcript and protein quantity, and it does not report on activity of proteins, which are regularly moderated through PTMs. Measurement of metabolite quantities reports on the culture status, as the metabolome is a product of the state of the cell, however, there are multiple confounding factors to relating metabolites to functional pathways such as how single metabolites can be quickly influenced through multiple metabolic pathways, meaning sampling time is highly important (47). This can be mitigated through the continual measurement of metabolites, which is achievable through on-line MS analysis (48, 49), or by more integrated *in silico* approaches such as metabolic flux analysis. Proteomics sample processing of bioreactors could be similarly streamlined as automated tissue sample preparation machines (50) and online digestion systems exist (51), however there may be a higher cost entry for this technology.

From global untargeted workflows, we were able to identify key proteins that are differentially regulated in CHO cells, which map onto existing understanding of CHO cell metabolism within bioreactors (22, 24). High expression of proteins such as LAMP1 and GSTM7 during the CHO cell stress phase suggests that the drop in viable cell numbers (Fig. 3 and Supplemental Fig. S2) may be driven by oxidative stress, in line with other studies (24, 52). Additional feed materials such as S-sulfocysteine have been shown to increase titers via specific anti-oxidant activity, so additional feed could be supplied in response to increase anti-oxidative response rather than preemptively (53). The increase in MCAT suggests that supplemental carnitine could be an ideal choice, considering the oxidative buffering capabilities of carnitine, its underlying support of the lactate switch and amino acid production, and that carnitine may be key for further increasing CHO production capabilities (42, 43). The entire quantitative proteomics discovery data sets provide a rich resource which could have further application, and while we select eight biomarkers, parallel reaction monitoring techniques on the latest available technology can push that number into the hundreds or even thousands of biomarkers on comparable time scales (54).

As the protein targets are representative of the key cellular processes occurring during fed batch bioreactors, a similar approach could be used to examine bioreactors under different run conditions, giving better information as to how the cells are responding to different growth conditions. For instance, within perfusion bioreactors, apoptotic-resistant cells undergo several exponential growth phases (55) or provide data for dynamic self-driving bioreactor cultivation (56). Other potential applications would be in cell line development. During the selection of stably transfected cell lines for the production of biotherapeutics, hundreds of cells may be automatically selected and grown into monoclonal colonies (57). Performing this targeted assay on both high and low producing clones may help identify biochemical reasons for this, aiding in the identification of gene targets for improving host cell productivity.

Previous applications of proteomics to bioprocessing of CHO cells, for example, from Betenbaugh and coworkers, have revolved around providing a deeper understanding of CHO cells (55, 58, 59, 60, 61, 62, 63, 64, 65, 66, 67). Fewer studies revolve around translational applications of the discovery data: Gao *et al.,* used existing knowledge of CHO cell production to create an MRM assay for lipases known to cause a decrease in product quality (68), and Albrecht *et al.*, translated discovery work (63) into an MRM assay, however, only targeted proteins involved in necrosis and apoptosis were examined (69). In contrast, our study targets an expanded set of cellular processes. Furthermore, while much research has been performed into creating an in-depth understanding of CHO cells under biomanufacturing conditions, due to the high genetic plasticity of CHO cell lines, it is imperative to understand specific production cell lines (70, 71).

The results here demonstrate the utility of applying discovery proteomics and translation to the biotechnology sector, with the identification of protein targets involved in the metabolic procession of CHO cells within a fed-batch bioreactor, and offer possible applications of this pipeline for the automated monitoring of bioreactor processes and applications for cell-line development. The relative quantities of these protein targets, which range from proliferation to oxidative stress and apoptosis, are indicative of the cellular status of the bioreactor and give a more in-depth understanding than gross biological phenotypes such as viable cell count. Further analysis of this dataset and others may yield more targets, giving exquisite understanding of the status of the bioreactor during production runs.

## Data Availability

The raw mass spectrometry data, spectral libraries, and analysis software parameters have been deposited to the ProteomeXchange consortium via the PRIDE repository with the dataset identifier PXD057984.

## Supplemental Data

This article contains supplemental data (18, 19, 20, 21).

## Declaration of Generative AI and AI-Assisted Technologies

No AI or AI-assisted technologies were used in this work.

## Conflict of interest

The authors declare the following financial interests/personal relationships which may be considered as potential competing interests: At the time of publication RH, JR and LP are employees of FUJIFILM Diosynth Biotechnologies.

## Supplemental Data

Reactome_analysis_worksheet
