# Supplementary material for: From Discovery to Delivery: A Rapid and Targeted Proteomics Workflow for Monitoring Chinese Hamster Ovary Biomanufacturing
Source: Mol Cell Proteomics. 2025 Jun 4;24(7):101011. doi: 10.1016/j.mcpro.2025.101011 (PMC12274832; doi:10.1016/j.mcpro.2025.101011)
Supplement: Supplemental Tables and Figures [file mmc2.docx]

**From Discovery to Delivery: A Rapid and Targeted Proteomics Workflow for Monitoring Chinese Hamster Ovary Biomanufacturing: Supplementary Information**

Charles Eldrid^1^*, Ellie Hawke^1^, Kathleen M. Cain^1^, Kate Meeson^1^, Joanne Watson^1^, Reynard Spiess^1^, Luke Johnston^2^, William Smith^1^, Matthew Russell^1^, Robyn Hoare^3^, John Raven^3^, Jean-Marc Schwartz^4^, Magnus Rattray^4^, Leon Pybus^3^, Alan Dickson^1^, Andrew Pitt^1^, Perdita Barran^1^*

^1^Manchester Institute of Biotechnology, University of Manchester, Princess St, Manchester, UK, M1 7DN; ^2^Institute of Quantitative Biology, Biochemistry & Biotechnology, School of Biological Sciences, University of Edinburgh, Edinburgh, EH9 3JW, UK; ^3^ FUJIFILM Diosynth Biotechnologies, Belasis Avenue, Billingham, UK, TS23 1LH; ^4^ Faculty of Biology, Medicine and Health, University of Manchester, Oxford Rd, Manchester, M13 9PL, UK

**Fed Batch Bioreactors:** The proprietary FUJIFILM Diosynth biotechnologies (FDB) CHO DG44-IgG-expressing cell line was cultured in a proprietary medium, FDB-MAP (FDB, Billingham, UK) supplemented with 175 nM MTX (Sigma) and 8mM L-glutamine (Sigma). The cell line was sub-cultured every 3-4 days in vented Erlenmeyer shake flasks (Corning, Amsterdam, Netherlands) at a seeding density of 0.5x106 cells/mL and maintained in a shaking cell culture incubator set at 125 rpm, 37 °C, 5% CO2 and >80% humidity.

Fed-batch bioreactor cultures were performed over a 14-day period in 2 L single-use Univessel’(R) (Sartorius) operated in dual mode. Cell cultures were performed in triplicate. Culture medium for 2 L bioreactor fed-batch cultures was JM-05B (FDB, Billingham, UK), that was at the start of culture supplemented with 8mM L-glutamine. Single use bioreactor vessels were filled with 1.2 L of medium and inoculated to a target seed density of 0.5x106 cells/mL. The pH was maintained at 7.0+/- 0.05 using carbon-dioxide gas sparging and base addition. The temperature was maintained at 37 °C through the bioreactor jacket. Dissolved oxygen was maintained at 40% of air saturation. The bioreactors were sampled immediately after inoculation and daily from day 2 for viable cell count (VCC), viability and extracellular metabolite concentrations. Samples for IgG analysis (~1 mL) were taken from day 3 onwards, and these medium samples were centrifuged (1000 g, 5 min) using a bench top micro-centrifuge and the resulting supernatants were transferred to a 1.5 mL microcentrifuge tube for storage prior to analyses. Bioreactor sampling preceded the daily bioreactor feeding, with cultures fed daily from day 2 onwards using a proprietary feeding regime.

**Proteomics Sample Preparation:** Cell pellets of 1E7 cells were defrosted and lysed using 50 µL of lysis buffer (5 % sodium dodecyl sulphate (SDS), 50 mM tetraethylammonium bromide (TEAB) pH 8.5 (Fisher Scientific, UK)) and were sonicated for 5 minutes in a bath sonicator. To remove DNA, 2 µL of Benzonase (Cambridge Bioscience Limited, UK) was added, and the samples were then sonicated again for another 5 minutes. The sample was then clarified by centrifugation at 13,000 RCF for 8 minutes, and the protein concentration of the lysate was calculated using the Pierce™ BCA Protein Assay Kit (ThermoFisher Scientific, UK) according to protocol, measured using a CLARIOstar (BMG Labtech, UK) plate reader. Lysate corresponding to 30 µg of protein were then taken forward and reduced in 5 mM TCEP (Cambridge Bioscience Limited, UK) for 15 minutes at 55 ˚C. The samples were then alkylated in 20 mM IAA (Fluorochem Limited, UK) for 1 hour in the dark at room temperature. The sample was acidified by the addition of phosphoric acid (VWR International Ltd, UK) to 2.5 % (v:v). The protein was then crashed through the addition of 165 µL of binding/wash buffer (100 mM TEAB in 90 % methanol) and applied to S-Trap™^1^ micro spin columns (VWR International, UK). The sample was washed three times with 150 µL of binding/wash buffer, and 1:10 ratio of trypsin gold (Promega, UK) to protein and incubated overnight at 37 ˚C in a thermomixer (Starlabs, UK). After incubation, peptides were then eluted with 50 µL of 50 mM TEAB, then 50 µL of 0.2 % formic acid, and then 50 µL of 50 % acetonitrile. The eluted solution was dried down before being resuspended in 0.1 % formic acid before MS analysis. Fractionated peptides for spectral library creation were prepared by applying 100 µg of peptides from ApolloX 221-107 cell pellets collected from maintenance sub-culture flasks (equivalent to exponential phase in bioreactor culture) to Pierce™ high pH reversed-phase peptide fractionation kit (ThermoFisher Scientific, UK).

**LC-MS:** 200 ng of sample were directly injected onto a nanoEase M/Z Peptide CSH C18 Column, 130Å, 1.7 µm, 300 µm X 150 mm (Waters Corp, UK) on a Acquity UPLC M-class system (Waters Corp, UK). Quality control (QC) injections of 23 ng of MassPREP enolase digest standard (Waters Corp, UK) every 15 injections, with blanks run every 5 injections. **ZenoToF 7600:** data were collected in positive mode using a 50 minute gradient at 2 µL/min (see Table S1), using an OptiFlow 50-200 µL Micro/MicroCal source. Autocalibration was performed every five injections. For DIA mode instrument parameters (Table S2), data was collected using 105 variable windows from 450 to 2050 *m/z* (Table S3).

| **Time (min)** | **A%** | **B%** | **Flowrate (uL/min)** |
| --- | --- | --- | --- |
| 0 | 97 | 3 | 2 |
| 3 | 97 | 3 | 2 |
| 32.5 | 75 | 25 | 2 |
| 40 | 62.5 | 40 | 2 |
| 41 | 5 | 95 | 2 |
| 43 | 5 | 95 | 2 |
| 44 | 97 | 3 | 2 |
| 50 | 97 | 3 | 2 |

**Table S1** LC gradient used on the Zeno ToF 7600, where A is 0.1% formic acid, and B is acetonitrile with 0.1% formic acid.

| **Parameter** | **Value** | **Parameter** | **Value** |
| --- | --- | --- | --- |
| Curtain Gas | 35 | TOF End Mass | 2050 |
| CAD gas | 7 | TOF MS Accumulation time | 0.1 s |
| Ion source gas 1 | 20 | Declustering potential | 80 V |
| Ion gas source 2 | 60 | Fragmentation mode | CID |
| Temperature | 225 °C | TOF MSMS start mass | 100 |
| Column Temperature | 50 °C | TOF MSMS end mass | 2000 |
| Spray Voltage | 5000 V | TOF MSMS Accumulation time | 0.018 s |
| TOF Start Mass | 400 | Total scan time | 2.394 s |

**Table S2** DIA instrument parameters for the ZenoTOF 7600

| **Window number** | **Start Da** | **End Da** | **CE (V)** | **Window Number** | **Start Da** | **End Da** | **CE (V)** |
| --- | --- | --- | --- | --- | --- | --- | --- |
| 0 | 400.0 | 406.5 | 21 | 50 | 659.5 | 666.5 | 34 |
| 1 | 405.5 | 412.5 | 21 | 51 | 665.5 | 672.5 | 34 |
| 2 | 411.5 | 418.5 | 22 | 52 | 671.5 | 678.5 | 34 |
| 3 | 417.5 | 424.5 | 22 | 53 | 677.5 | 684.5 | 35 |
| 4 | 423.5 | 430.5 | 22 | 54 | 683.5 | 690.5 | 35 |
| 5 | 429.5 | 436.5 | 22 | 55 | 689.5 | 696.5 | 35 |
| 6 | 435.5 | 442.5 | 23 | 56 | 695.5 | 702.5 | 35 |
| 7 | 441.5 | 448.5 | 23 | 57 | 701.5 | 708.5 | 36 |
| 8 | 447.5 | 454.5 | 23 | 58 | 707.5 | 714.5 | 36 |
| 9 | 453.5 | 459.5 | 24 | 59 | 713.5 | 720.5 | 36 |
| 10 | 458.5 | 464.5 | 24 | 60 | 719.5 | 726.5 | 37 |
| 11 | 463.5 | 469.5 | 24 | 61 | 725.5 | 732.5 | 37 |
| 12 | 468.5 | 474.5 | 24 | 62 | 731.5 | 738.5 | 37 |
| 13 | 473.5 | 479.5 | 25 | 63 | 737.5 | 744.5 | 37 |
| 14 | 478.5 | 484.5 | 25 | 64 | 743.5 | 750.5 | 38 |
| 15 | 483.5 | 489.5 | 25 | 65 | 749.5 | 756.5 | 38 |
| 16 | 488.5 | 494.5 | 25 | 66 | 755.5 | 763.5 | 38 |
| 17 | 493.5 | 499.5 | 25 | 67 | 762.5 | 770.5 | 39 |
| 18 | 498.5 | 504.5 | 26 | 68 | 769.5 | 777.5 | 39 |
| 19 | 503.5 | 509.5 | 26 | 69 | 776.5 | 784.5 | 39 |
| 20 | 508.5 | 514.5 | 26 | 70 | 783.5 | 791.5 | 40 |
| 21 | 513.5 | 519.5 | 26 | 71 | 790.5 | 798.5 | 40 |
| 22 | 518.5 | 524.5 | 27 | 72 | 797.5 | 805.5 | 40 |
| 23 | 523.5 | 529.5 | 27 | 73 | 804.5 | 812.5 | 41 |
| 24 | 528.5 | 534.5 | 27 | 74 | 811.5 | 819.5 | 41 |
| 25 | 533.5 | 539.5 | 27 | 75 | 818.5 | 826.5 | 42 |
| 26 | 538.5 | 544.5 | 28 | 76 | 825.5 | 834.5 | 42 |
| 27 | 543.5 | 549.5 | 28 | 77 | 833.5 | 842.5 | 42 |
| 28 | 548.5 | 554.5 | 28 | 78 | 841.5 | 850.5 | 43 |
| 29 | 553.5 | 559.5 | 28 | 79 | 849.5 | 858.5 | 43 |
| 30 | 558.5 | 564.5 | 29 | 80 | 857.5 | 867.5 | 44 |
| 31 | 563.5 | 569.5 | 29 | 81 | 866.5 | 876.5 | 44 |
| 32 | 568.5 | 574.5 | 29 | 82 | 875.5 | 885.5 | 44 |
| 33 | 573.5 | 579.5 | 29 | 83 | 884.5 | 894.5 | 45 |
| 34 | 578.5 | 584.5 | 30 | 84 | 884.5 | 903.5 | 45 |
| 35 | 583.5 | 589.5 | 30 | 85 | 902.5 | 944.6 | 46 |
| 36 | 588.5 | 594.5 | 30 | 86 | 943.6 | 999.6 | 48 |
| 37 | 593.5 | 599.5 | 30 | 87 | 999.1 | 1074.6 | 51 |
| 38 | 598.5 | 604.5 | 31 | 88 | 1074.1 | 1149.6 | 53 |
| 39 | 603.5 | 609.5 | 31 | 89 | 1149.1 | 1224.6 | 58 |
| 40 | 608.5 | 614.5 | 31 | 90 | 1224.1 | 1299.6 | 62 |
| 41 | 613.5 | 619.5 | 31 | 91 | 1299.1 | 1374.6 | 65 |
| 42 | 618.5 | 624.5 | 32 | 92 | 1374.1 | 1449.6 | 69 |
| 43 | 623.5 | 629.5 | 32 | 93 | 1449.1 | 1524.6 | 73 |
| 44 | 628.5 | 634.5 | 32 | 94 | 1524.1 | 1599.6 | 76 |
| 45 | 633.5 | 639.5 | 32 | 95 | 1599.1 | 1674.6 | 80 |
| 46 | 638.5 | 644.5 | 33 | 96 | 1674.1 | 1774.1 | 80 |
| 47 | 643.5 | 649.5 | 33 | 97 | 1773.6 | 1873.6 | 81 |
| 48 | 648.5 | 654.5 | 33 | 98 | 1873.1 | 1973.1 | 81 |
| 49 | 653.5 | 660.5 | 33 | 99 | 1972.6 | 2050.0 | 82 |

**Table S3** DIA SWATH window ranges with associated collision energies in volts.

| **Parameter** | **Value** | **Parameter** | **Value** |
| --- | --- | --- | --- |
| Maximum candidate ions | 20 | Exclude former candidate ions | True |
| Intensity threshold exceeds | 100 | Exclusion time | 30 s |
| Dynamic background subtract | True | Exclusion occurrences | 2 |

**Table S4** Additional DDA parameters for the ZenoTOF 7600

| **Charge** | **Slope** | **Intercept** |
| --- | --- | --- |
| 2 | 0.049 | -1 |
| 3 | 0.048 | -2 |
| 4 | 0.050 | -2 |
| 5 | 0.050 | -2 |

**Table S5** Dynamic collision energy calculated for MSMS by CE = (slope) * (m/z) + (intercept) for CID in the ZenoTOF 7600

**Cyclic IMS QToF:** data were collected using a 100 minute gradient (**Table S6**), with mobility, using system settings adapted from Nagy, K. *et al.*^2^ (**Table S7** and **Table S8**) with a mass range of 50-2000 m/z.

| **Time (min)** | **A%** | **B%** | **Flowrate (uL/min)** |
| --- | --- | --- | --- |
| 0.00 | 97 | 3.00 | 0.6 |
| 3.00 | 97 | 3.00 | 0.6 |
| 65.00 | 70.0 | 30.00 | 0.6 |
| 78.00 | 55.0 | 45.00 | 0.6 |
| 80.00 | 5 | 95.00 | 0.6 |
| 85.10 | 5 | 95.00 | 0.6 |
| 90.0 | 97 | 3.00 | 0.6 |
| 100.0 | 97 | 3.00 | 0.6 |

**Table S6** LC gradient used on the Cyclic IMS QToF, where A is 0.1% formic acid, and B is acetonitrile with 0.1% formic acid.

| **Parameter** | **Value** | **Parameter** | **Value** |
| --- | --- | --- | --- |
| Capillary | 3.5 kV | Transfer exit | 15 V |
| Cone | 40 V | Trap height | 4 V |
| Source offset | 10 V | Pushes per bin | 5 |
| Source temperature | 100 °C | Static Twave | 15 V |
| Desolvation temperature | 250 °C | Trapping height | 10 V |
| Reference capillary | 3.5 kV | Transfer entrance | 2 V |
| Trap entrance | 2 V | Twave start height | 15 V |
| Trap Bias | 2 V | Twave end height | 35 V |
| Trapping height | 10 V | Twave ramp rate | 2.5 V |
| Transfer entrance | 2 V | Transfer CE ramp start | 15 V |
| Transfer gradient | 4 V | Transfer CE ramp end | 40 V |
| Static offset | 180 V |  |  |

**Table S7** HDMSe data collection instrument parameters for the Cyclic IMS QToF

| **Function** | **Time** | **Pre Array Gradient** | **Pre Array Bias** | **Entrance** | **Exit** | **Offset** | **Wave Amplitde** | **Post Array Gradient** | **Post Array Bias** |
| --- | --- | --- | --- | --- | --- | --- | --- | --- | --- |
| Inject | 10 | 85 | 70 | 10 | 50 | 45 | 2 | 35 | 10 |
| Separate | 2 | 85 | 70 | 30 | 30 | 70 | 0 | 35 | 10 |
| Eject and Acquire | - | 85 | 70 | 50 | 2 | 45 | 25 | 35 | 10 |

**Table S8** Cyclic mobility sequence

**MRM Assay**: To develop a tier 3 multiple reaction monitoring assay ‘quantotypic’ target peptides detected in the discovery data sets for the respective targeted proteins which were found to be unique to the mammalian Uniprot database and did not contain residues such as methionine or asparagine. Synthetic target peptides were purchased from JPT Peptide Technologies (DE), and prepared in 0.1% formic acid, and used for method development. Data were collected using a 15 minute gradient (**Table S9** Focus LC gradient used on the Xevo TQS**Table S9**) on a Xevo TQS (Waters, UK) triple quad mass spectrometer, for instrument parameters see **Table S10**. For targeted method development initial instrument acquisition file creation, collision energy optimization and fragment selection were performed using Skyline (v23.1.0.455). For transition parameters see **Table S11**. Calculation of peak areas for each transition were performed in Skyline.

| **Time (min)** | **A%** | **B%** | **Flowrate (mL/min)** |
| --- | --- | --- | --- |
| 0 | 95 | 5 | 0.5 |
| 2 | 95 | 5 | 0.5 |
| 2.1 | 90 | 10 | 0.5 |
| 12 | 73 | 27 | 0.5 |
| 12.1 | 5 | 95 | 0.5 |
| 13 | 5 | 95 | 0.5 |
| 13.1 | 95 | 5 | 0.5 |
| 13.2 | 95 | 5 | 0.5 |
| 13.4 | 5 | 95 | 0.5 |
| 13.5 | 5 | 95 | 0.5 |
| 13.6 | 95 | 5 | 0.5 |
| 13.7 | 95 | 5 | 0.5 |
| 13.8 | 5 | 95 | 0.5 |
| 13.9 | 5 | 95 | 0.5 |
| 14 | 95 | 5 | 0.5 |
| 14.1 | 95 | 5 | 0.5 |
| 14.2 | 5 | 95 | 0.5 |
| 14.3 | 95 | 5 | 0.5 |
| 15 | 95 | 5 | 0.5 |

**Table S9** Focus LC gradient used on the Xevo TQS, where A is 0.1% formic acid, and B is acetonitrile with 0.1% formic acid.

| **Parameter** | **Value** | **Parameter** | **Value** |
| --- | --- | --- | --- |
| Capillary | 3.0 kV | Cone gas flow | 150 L/Hr |
| Cone | 40 V | Desolvation gas flow | 800 L/Hr |
| Source offset | 50 V | Collision Gas Flow | 0.15 L/Hr |
| Source temperature | 150 °C | Nebuliser gas flow | 7.00 L/Hr |
| Desolvation temperature | 600 °C | Dwell | 0.09 |

**Table S10** Instrument parameters for data collection on the Xevo TQS


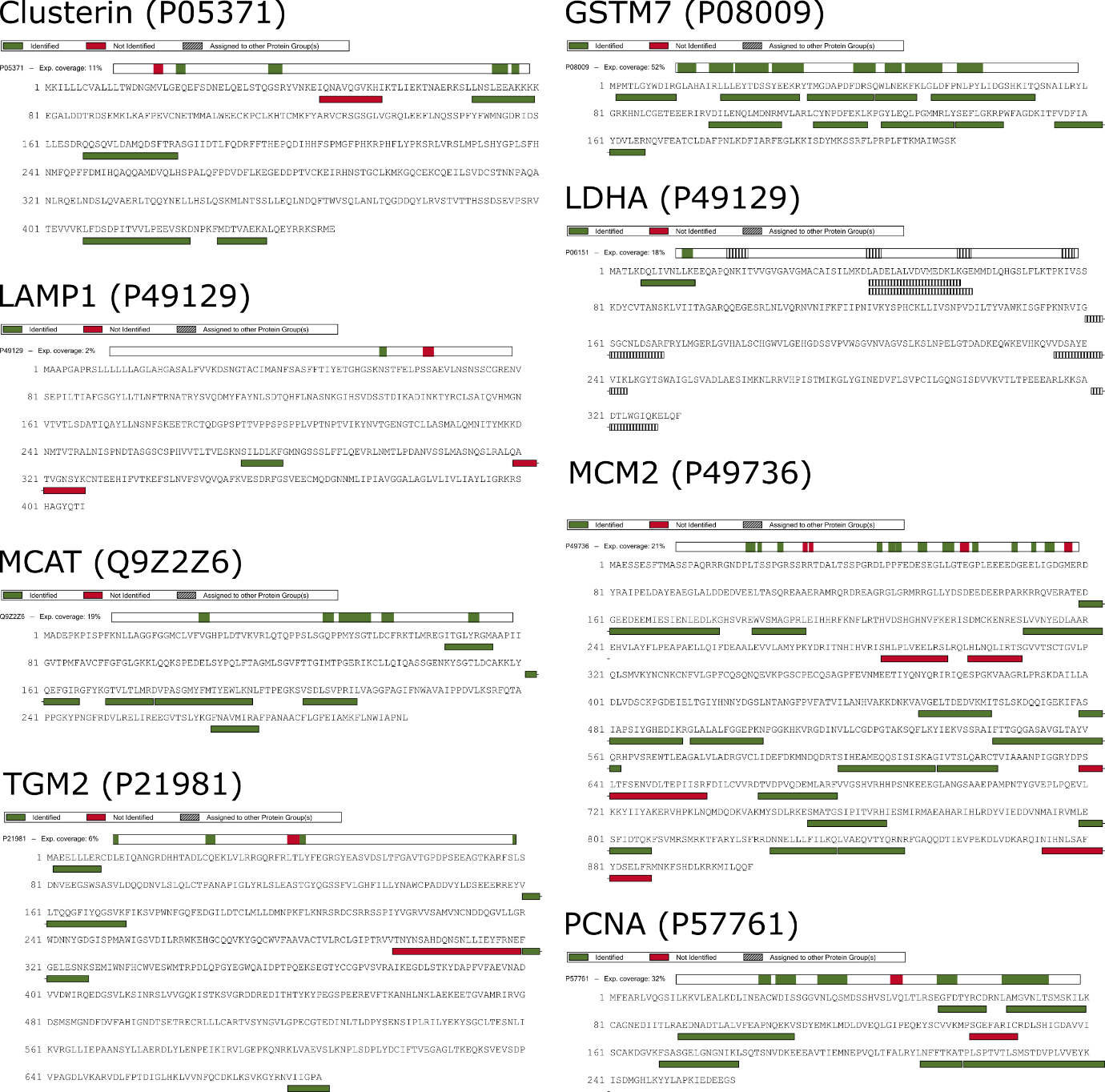


**Figure S1**: Peptide coverage of proteins selected for MRM analysis. Green: identified for protein group, red: not identified, striped: matched to different protein group

| **Uniprot ID** | **Protein** | **Peptide** | **Fragment** | **Q1** | **Q2** | **Collision Energy (eV)** |
| --- | --- | --- | --- | --- | --- | --- |
| P49129 | LAMP1 | NSILDLK++ | y2+ | 401.74 | 260.20 | 16 |
|  |  |  | y3+ | 401.74 | 375.22 | 17 |
|  |  |  | y4+ | 401.74 | 488.31 | 15 |
| P08009 | GSTM7 | LYSEFLGK++ | y2+ | 478.76 | 204.13 | 17 |
|  |  |  | b2+ | 478.76 | 277.15 | 12 |
|  |  |  | y6+ | 478.76 | 680.36 | 13 |
| Q9Z2Z6 | MCAT | SVSDLSVPR++ | b2+ | 480.26 | 187.11 | 15 |
|  |  |  | y4+ | 480.26 | 458.27 | 16 |
|  |  |  | y7+ | 480.26 | 773.42 | 15 |
| P49736 | MCM2 | AGIVTSLQAR++ | b3+ | 508.30 | 242.15 | 21 |
|  |  |  | y6+ | 508.30 | 675.38 | 22 |
|  |  |  | y7+ | 508.30 | 774.45 | 20 |
| P06151 | LDHA | DQLIVNLLK++ | b2+ | 528.33 | 244.09 | 17 |
|  |  |  | y5+ | 528.33 | 586.39 | 17 |
|  |  |  | y7+ | 528.33 | 812.56 | 15 |
| P05371 | CLUS | SLLNSLEEAK++ | b2+ | 552.30 | 201.12 | 16 |
|  |  |  | y6+ | 552.30 | 676.35 | 21 |
|  |  |  | y8+ | 552.30 | 903.48 | 16 |
| P57761 | PCNA | FSASGELGNGNIK++ | b2+ | 647.33 | 235.11 | 28 |
|  |  |  | y6+ | 647.33 | 602.33 | 19 |
|  |  |  | y11+ | 647.33 | 1059.54 | 21 |
| P21981 | TGM2 | NVIIGPA+ | y2+ | 683.41 | 187.11 | 29 |
|  |  |  | b3+ | 683.41 | 327.20 | 22 |
|  |  |  | y4+ | 683.41 | 357.21 | 21 |

**Table S11** MRM settings for the peptide assay, as optimised by Skyline

**Data Analysis** Proteomics data were analysed using Spectronaut v18.4 (Biognosys)^3^ using BGS factory settings in a peptide-centric fashion. A spectral library was created from fractionated peptide data dependent acquisition using the Pulsar library creation method in Spectronaut (v 18.4.231017.55695), generated from an *in-silico* library based on reviewed mammalian protein sequences from Uniprot containing 67,711 entries downloaded on 2023-09-04. *In silico* peptides where generated using Trypsin/P digest rules with peptide length between 7-52, with a maximum of 2 missed cleavages, fixed modifications of carbamidomethyl cysteine modifications and variable modifications of acetylated N-terminus and oxidation of methionine with a maximum number of 5 variable modifications. A false discovery rate of 1 % was used for both peptides and proteins. Data independent acquisition (DIA) data were searched using the DIA+ method, with both the spectral library and mammalian sequence database used for spectral library creation using the same *in silico* peptide parameters. Peptides were identified using a dynamic mass tolerance, with a 1 % false discovery rate based on mutated decoy sequences. Q-value of the precursor and the protein cut-off were set to 0.05, quantification was based on MS2 area, with the normalisation strategy set to “automatic”. Full details of the analysis pipeline, spectral libraries, and raw data are available through the ProteomeXchange consortium via the PRIDE repository with the dataset identifier PXD057984. Heatmaps were produced in Spectronaut, and further analysis was performed using Python 3.7.

Uniprot accessions were mapped to gene names using the Uniprot ID mapper tool (<https://www.uniprot.org/id-mapping>). Missing gene names were mapped manually. Statistically enriched pathways were identified using were mapped onto human (*Homo sapiens*), rat (*Rattus novergicus*) and mouse (*Mus musculus*) pathways in Reactome (on 2024-08-13), with a pathway threshold of p-value≤0.05.^4^

# Supplementary Data

**Targeted Analysis**


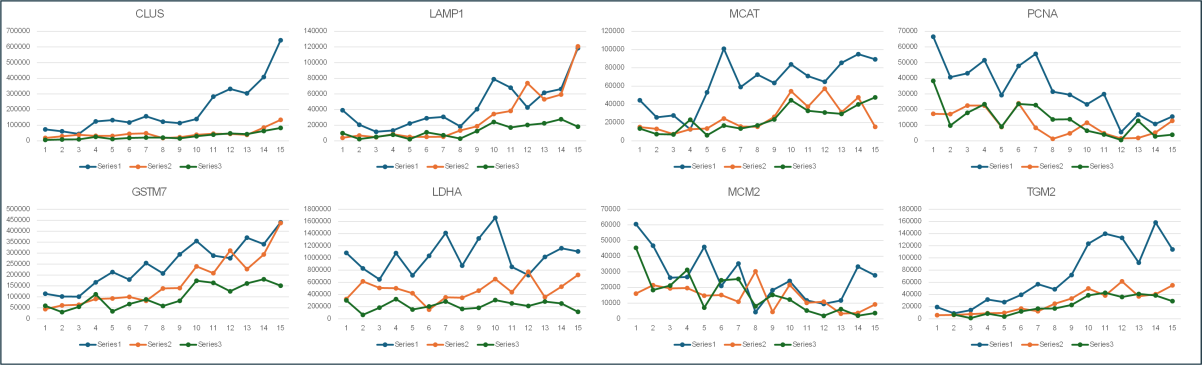


**Figure S2** Absolute measured intensity of quantified peptides from triplicate bioreactors, showing biological variation


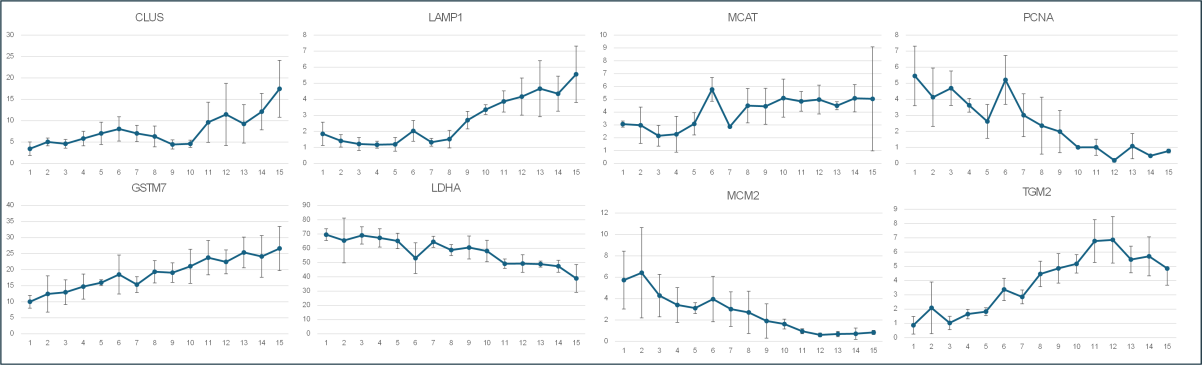


**Figure S3** Averaged ratio area under the curve per bioreactor

# Bibliography

1. HaileMariam, M. *et al.* S-Trap, an Ultrafast Sample-Preparation Approach for Shotgun Proteomics. *J. Proteome Res.* **17**, 2917–2924 (2018).

2. Nagy, K., Gellén, G., Papp, D., Schlosser, G. & Révész, Á. Optimum collision energies for proteomics: The impact of ion mobility separation. *J. Mass Spectrom.* **n/a**, e4957.

3. Bruderer, R. *et al.* Extending the Limits of Quantitative Proteome Profiling with Data-Independent Acquisition and Application to Acetaminophen-Treated Three-Dimensional Liver Microtissues. *Mol. Cell. Proteomics* **14**, 1400–1410 (2015).

4. Fabregat, A. *et al.* Reactome pathway analysis: a high-performance in-memory approach. *BMC Bioinformatics* **18**, 142 (2017).
